# Classification and application of metal-based nanoantioxidants in medicine and healthcare

**DOI:** 10.3762/bjnano.15.36

**Published:** 2024-04-12

**Authors:** Nguyen Nhat Nam, Nguyen Khoi Song Tran, Tan Tai Nguyen, Nguyen Ngoc Trai, Nguyen Phuong Thuy, Hoang Dang Khoa Do, Nhu Hoa Thi Tran, Kieu The Loan Trinh

**Affiliations:** 1 Applied Biology Center, School of Agriculture and Aquaculture, Tra Vinh University, Tra Vinh City 87000, Vietnam; 2 College of Korean Medicine, Gachon University, 1342 Seongnam-daero, Sujeong-gu, Seongnam-si 13120, Republic of Korea; 3 Department of Materials Science, School of Applied Chemistry, Tra Vinh University, Tra Vinh City 87000, Vietnam; 4 NTT Hi-Tech Institute, Nguyen Tat Thanh University, Ward 13, District 04, Ho Chi Minh City 70000, Vietnam; 5 Faculty of Materials Science and Technology, University of Science, Ho Chi Minh City, Vietnam; 6 Vietnam National University Ho Chi Minh City, Vietnam; 7 BioNano Applications Research Center, Gachon University, 1342 Seongnam-daero, Sujeong-gu, Seongnam-si 13120, Republic of Korea

**Keywords:** antioxidant nanomaterial, healthcare, medicine, nanotechnology, oxidative stress

## Abstract

Antioxidants play an important role in the prevention of oxidative stress and have been widely used in medicine and healthcare. However, natural antioxidants have several limitations such as low stability, difficult long-term storage, and high cost of large-scale production. Along with significant advances in nanotechnology, nanomaterials have emerged as a promising solution to improve the limitations of natural antioxidants because of their high stability, easy storage, time effectiveness, and low cost. Among various types of nanomaterials exhibiting antioxidant activity, metal-based nanoantioxidants show excellent reactivity because of the presence of an unpaired electron in their atomic structure. In this review, we summarize some novel metal-based nanoantioxidants and classify them into two main categories, namely chain-breaking and preventive antioxidant nanomaterials. In addition, the applications of antioxidant nanomaterials in medicine and healthcare are also discussed. This review provides a deeper understanding of the mechanisms of metal-based nanoantioxidants and a guideline for using these nanomaterials in medicine and healthcare.

## Introduction

Reactive oxygen species (ROS) play an important role in proper cellular functions and adaptation. However, an excess of free ROS in biological systems can lead to oxidative stress-related diseases such as inflammatory disorders, neurological diseases, aging-related diseases, and cancers [1–2]. The human body naturally defends itself against oxidative stress by using antioxidant biomolecules. With the excellent ROS scavenging effect, antioxidants significantly contribute to the balance of ROS and protect the human body from free radicals, which are produced either by normal metabolism or an effect of exposure to external factors [3–4]. However, the natural antioxidants in our body do not always work efficiently because ROS are so pervasive. Although antioxidant supplements from natural sources such as plants and animals are considered an effective strategy to combat oxidative stress, antioxidants from natural sources still have some notable limitations. These are low stability, low bioavailability, difficult long-term storage, and high cost of large-scale production [5–6].

Recently, nanomaterials have emerged as a promising strategy to overcome the limitations of natural antioxidants because of their high stability, easy storage, time effectiveness, and low cost. Also, progress in nanotechnology enables us to easily control size, morphology, surface coating, and chemical configuration, which are highly related to the antioxidant activities. The integration of biomedicine and nanotechnology for developing new types of antioxidants has witnessed a technological breakthrough that resulted in extraordinary progress in the pharmaceutical and biotechnology industries [7–8]. Moreover, many categories of nanomaterials have shown greater antioxidant activity and more resistance to severe environments than the antioxidants originating from plants and animals. More interestingly, through nanoencapsulation and nanodelivery, antioxidant nanomaterials improve the pharmacokinetics of natural antioxidants by preventing their degradation under stress conditions [9–10].

Antioxidant nanomaterials can be synthesized from carbon-based compounds, polymeric compounds, and metal-based compounds. Metal-based nanoantioxidants exhibit strong reactivity because there are atoms with unpaired electrons on the surface. Therefore, metal-based nanoantioxidants have a significant advantage over natural antioxidants as well as other types of nanoantioxidants with lower reactivity and stability. As a result, metal-based nanoantioxidants have gathered significant attention from scientists. Many efforts have been made to develop new metal-based nanoantioxidants, understand the mechanisms of action, and expand their applications, especially in medicine and healthcare. For example, the question of why nanoparticles with a majority of Ce^3+^ on the surface have stronger antioxidant activity than those with Ce^4+^ has recently been answered by Dutta and co-workers [11]. Ce^3+^ nanoparticles have a single 4f^1^ electron, which can be easily given up in a reaction with ROS. In contrast, Ce^4+^ nanoparticles have an octet-filled xenon configuration leading to less chemical activity. Understanding the mechanisms of metal-based nanoantioxidants is vitally important because it helps to rationally design and safely apply these nanomaterials for human healthcare, which strictly require assessment regarding quality control, safety, and efficacy. Many in vitro and in vivo assessments have been reported to prove the potential of metal-based nanomaterials for scavenging free radicals. FeO nanoparticles have 81% DPPH inhibition and exhibit in vivo hepatoprotective properties in laboratory mice [12]. The combination of copper and cerium in nanoparticle structures enhances antioxidant activity through a synergistic effect. CuCe nanoparticles have been demonstrated for therapeutic effects in ischemic vascular diseases [13].

Current review articles have been published mainly focusing on the general mechanisms of the nanoantioxidants and their applications in various fields [14–16]. In addition to these works, this review tries to take a deeper look at nanoantioxidants and only focuses on the mechanisms of metal-based nanoantioxidants and their application in medicine and healthcare in the last ten years. First, we summarize some novel metal-based nanoantioxidants and classify them into two main categories, namely chain-breaking and preventive antioxidant nanomaterials. Some representative examples of metal-based nanoantioxidants of each type are described focusing on the mechanism of action, antioxidant activities, and individual properties. Second, the application of metal-based nanoantioxidants in medicine and healthcare is carefully discussed in order to demonstrate the potential of nanotechnology, especially for reducing oxidative stress.

## Review

### Metal-based nanomaterials exhibiting antioxidant activities

#### Preventive metal-based nanoantioxidants

**Figure 1:**
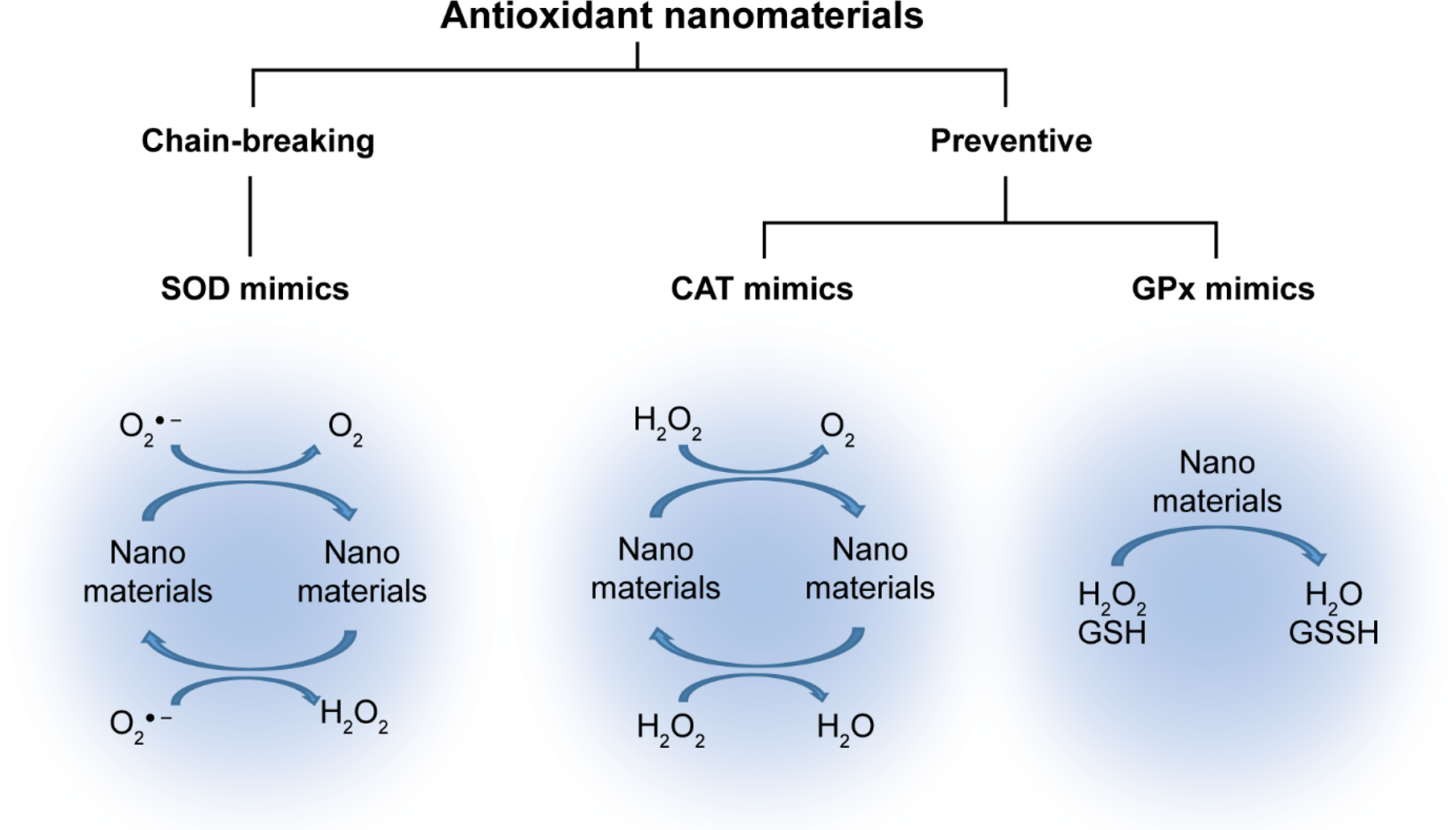
Classification of antioxidant nanomaterials.

Along the evolution, the human body has naturally developed a complex antioxidant defense system, which uses endogenous non-enzymatic and enzymatic biomolecules. With the assistance of co-factors, these biomolecules can effectively neutralize ROS or precursors of ROS to reduce oxidative damage to body tissues. As the first category, preventive antioxidants can eliminate agents involved in the initiation of oxidative reactions and, thus, prevent oxidative damage before an oxidative reaction can occur (Figure 1). Preventive antioxidants are also known as the first-line defense antioxidants because they efficiently neutralize or eliminate any potential molecule involved in free radical development. Preventive antioxidants prevent the formation of free radicals through several pathways such as chelating transition metals, quenching singlet oxygens, decomposing hydrogen peroxides, and deactivating superoxides without generating active radicals [17–19]. Transition metals such as Fe^2+^ and Cu^2+^ initiate a Fenton reaction in the presence of hydrogen peroxide or organic hydroperoxide (ROOH) to produce ROS. In nature, biological systems commonly use catalase (CAT) and glutathione peroxidase (GPx) as preventive antioxidants to degrade hydrogen peroxide, which is a precursor of ROS. CAT, containing a heme active site, and GPx, containing a selenium active site, can decompose hydrogen peroxides into harmless compounds (e.g., O_2_ and H_2_O). Although CAT and GPx show great potential for reducing oxidative stress, the disadvantages of natural enzymes such as low stability and little flexibility can limit their applications, especially in medicine and healthcare. Along with significant advances in nanotechnology, nanomaterials mimic natural antioxidant enzymes providing excellent properties such as high stability, low cost, and flexibility to improve the limitations of natural antioxidant enzymes.

**CAT mimics:** Nanomaterials exhibiting CAT activity mainly employ the properties of transition metals and metal oxides (e.g., cobalt, iron, cerium, and gold), which can generate a cycle of reduction and oxidation stages [20–23]. Among these metal oxides, cerium oxide-based nanomaterials have been deeply studied with regard to the mechanisms of CAT activity. Cerium-based nanomaterials exhibit CAT activity through the ability to decompose H_2_O_2_ into O_2_ and H_2_O. Cerium oxide consists of mixed valence stages of Ce^3+^ (reduced) and Ce^4+^ (fully oxidized), which allows for the generation of redox cycles for exhibiting CAT activity in the presence of H_2_O_2_. The redox cycles start when H_2_O_2_ binds to two Ce^4+^, reducing two Ce^4+^ into two Ce^3+^ while H_2_O_2_ is decomposed into O_2_ and two H^+^. Subsequently, two Ce^3+^ decompose the second H_2_O_2_ and are oxidized into two Ce^4+^ [24–25]. The redox cycle of Ce^4+^/Ce^3+^ for H_2_O_2_ decomposition can be described as follows:


[1] $$2{\text{Ce}}^{4+}+{\text{H}}_{2}{\text{O}}_{2}\to 2{\text{Ce}}^{3+}+{\text{O}}_{2}+2{\text{H}}^{+}$$

[2] $$2{\text{Ce}}^{3+}+{\text{H}}_{2}{\text{O}}_{2}+2{\text{H}}^{+}\to 2{\text{H}}_{2}\text{O}+2{\text{Ce}}^{4+}$$

**Figure 2:**
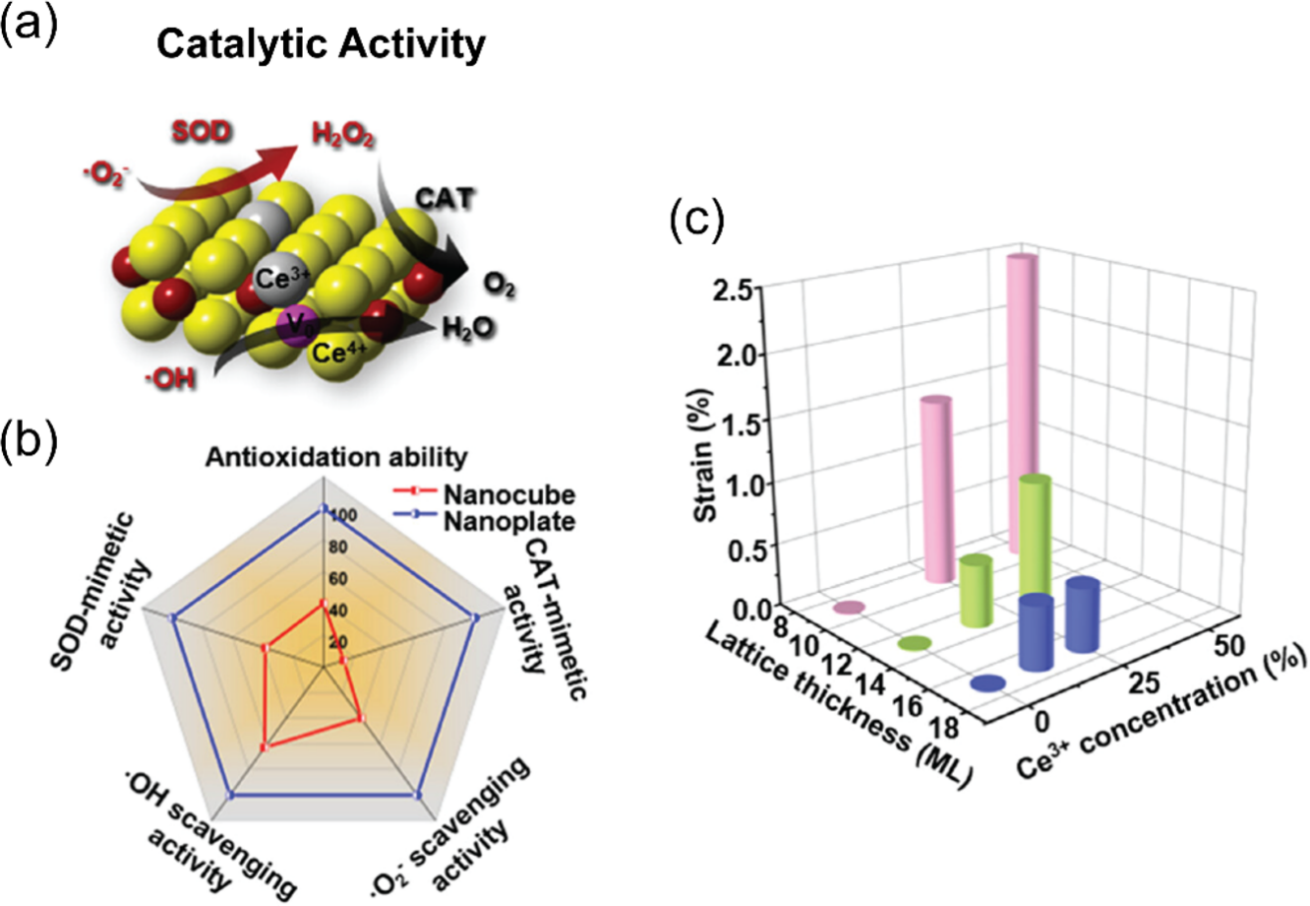
Multi-antioxidant activities of ceria nanoplates. (a) Schematic illustration of cascade antioxidant activity of ceria nanozyme with enzyme-mimetic and ROS scavenging properties. (b) Radar map of cascade antioxidant activity of the ceria nanozyme with enzyme-mimetic and ROS scavenging properties. (c) 3-Dimensional diagram of the relationship between lattice thickness, Ce^3+^ concentration, and the resulting strain in ceria with specific surface orientation (100). Figure 2 was adapted with permission from [32]. Copyright 2023 American Chemical Society. This content is not subject to CC BY 4.0.

It has been reported that the CAT activity of cerium oxide nanoparticles is highly affected by the Ce^3+^/Ce^4+^ ratio [26–27]. Several studies demonstrated that fewer Ce^3+^ atoms on the surface enhance the CAT activity of cerium oxide. Ce^4+^ shows greater H_2_O_2_ decomposition activity than Ce^3+^ [28–31]. Figure 2 shows the property of ceria nanoplates with multi-antioxidant activities and the relationship between lattice thickness, Ce^3+^ concentration, and the resulting strain in ceria with specific surface orientation (100) [32]. Similarly, a large number of different metal-based nanomaterials have been investigated for antioxidant activities. Singh et al. investigated the effect of the Mn^3+^/Mn^2+^ ratio on CAT activity [33]. Oxidizing Mn_3_O_4_ with NaIO_4_ yielded a high Mn^3+^/Mn^2+^ ratio and enhanced CAT activity compared with that of non-oxidized Mn_3_O_4_. Ten different iron oxide-based nanoantioxidants including 6-line ferrihydrite, 2-line ferrihydrite, goethite, akageneite, feroxyhyte, hematite, magnetite, maghemite, schwertmannite, and lepidocrocite were compared regarding CAT activity [34]. The highest CAT activity was shown by 2-line ferrihydrite, followed by 6-line ferrihydrite and feroxyhyte. Iron-based nanomaterials with more hydroxy groups in the stoichiometric composition generally tend to exhibit higher CAT activity because hydroxy groups enhance ion exchange on the surface of nanomaterials by providing active sites [35–36]. The CAT-like activity was also investigated in copper-based nanomaterials by Gao’s group [37]. The Michaelis–Menten constant (*K*_m_) and maximum initial velocity (*V*_max_*)* values showed that copper-doped hollow carbon spheres had an eightfold higher CAT-like activity than pure carbon nanozymes. The oxidation state of copper may play a more important role regarding CAT-like activity than copper content and size.

The enzyme activity of natural CAT highly depends on the three-dimensional (3D) structure of the protein. A significant decrease or even loss of CAT activity can be caused already by small changes in the protein conformation and structure. As a matter of fact, cellular microenvironments have different pH values and temperatures depending on the part and physiological status of the body. Thus, natural CAT usually suffers from low stability and is sensitively influenced by the environment. Several reports investigated the effect of pH and temperature on CAT activity as well the stability of CAT-mimicking nanomaterials. For example, ceria nanoparticles maintained a relative activity of approximately 100% at 20–70 °C, while natural CAT showed a significant decrease in relative activity at over 40 °C [38]. The decrease of catalytic activity of natural CAT at temperatures above 40 °C is caused by protein denaturation at high temperatures. Impressively, Co_3_O_4_ nanomaterials with different morphologies (nanoplates, nanorods, and nanocubes) exhibited the highest relative activity at very high pH (pH 9) and temperature (90 °C) [39]. Similar results were also reported for platinum nanoparticles whose H_2_O_2_ decomposition increased with increasing pH values (up to 11) and temperatures (up to 90 °C) [40]. The activity of natural CAT can also be improved by immobilizing it on nanomaterials. Immobilized CAT on Cu(II) nanofibers maintained approximately half of its catalytic activity after incubation at 60 °C for 5 h, while free CAT lost nearly 90% of the catalytic activity under the same conditions [41].

**GPx mimics:** GPx protects the body from oxidative stress by participating in the conversion of H_2_O_2_ into H_2_O. GPx requires the presence of glutathione (GSH) to function properly [42]. Similarly, GPx-mimicking nanomaterials use two GSH molecules as cofactors to generate redox cycles for H_2_O_2_ degradation. In GPx-mimicking V_2_O_5_ nanocrystals, H_2_O_2_ first binds to the surface of V_2_O_5_ converting V=O into V-peroxido intermediates. The V-peroxido intermediates then react with GSH to V−OH. Subsequently, V−OH recovers to V=O by reacting with another H_2_O_2_ in the presence of GSH [43–45]. Recently, Liu et at. constructed a trimetallic alloy nanozyme of gold, copper, and platinum, which exhibits multiple enzyme activities including that of GPx [46]. In this nanocomposite, platinum and copper sites are responsible for the adsorption, activation, and dissociation of the reactants, while the desorption occurs at the gold sites. The combination of multiple metals significantly improves the catalytic efficiency through synergistic effects. The nanocomposites not only disrupt the redox homeostasis in tumors through a series of cascade reactions but also establish cyclic regeneration of relevant substrates. Another study combined MnO_2_ nanoparticles with bovine serum albumin (BSA) to obtain GPx activity [47]. The in vitro results showed excellent biocompatibility of the MnO_2_-BSA nanoparticles and increased the survival rate of cells under high H_2_O_2_ concentration. In vivo results of another experiment demonstrated the ROS scavenging ability of Au@Cu_2_O heterostructures in a zebrafish model, which is an organism with morphological and physiological functions similar to those of humans [48]. The heterostructures increase surface electron deficiency, redox couples, and oxygen vacancies through an intrinsic electric field and lattice mismatch at the metal–semiconductor interface. Thus, a high level of oxygen vacancies enhances the adsorption and activation of oxygen-containing ROS, that is, H_2_O_2_ and O_2_^•–^.

#### Chain-breaking metal-based nanoantioxidants

Chain-breaking antioxidants slow down or stop oxidation reactions after they begin. Chain-breaking antioxidants can directly trap, scavenge, or convert ROS into more stable and nonradical products and, thus, compete with the propagation reactions. In other words, ROS tend to react with chain-breaking antioxidants more rapidly than with the substrate [8,49]. Phenolic compounds are among the most effective chain-breaking antioxidants that appear in nature and can also be synthesized. For example, α-tocopherol (vitamin E) is a chain-breaking antioxidant appearing in low-density lipoprotein and human blood plasma contributing to the decrease of oxidative stress. Its mechanism of action relies on the ability to transfer the phenolic H atom to a peroxyl radical (ROO^•^) much more rapidly than the propagation reactions [50–53]. Despite the promising application of chain-breaking antioxidants for scavenging ROS, most chain-breaking antioxidants have unsaturated bonds and strong antioxidant activity, which is greatly affected by pH variations, heat, light, and the amount of metal and oxygen. Moreover, chain-breaking antioxidants showed limited stability during long-term storage [54–56].

Relying on the mechanism of chain-breaking antioxidants, nanomaterials can be synthesized to directly react with ROS after the oxidative reaction begins. Chain-breaking antioxidant nanomaterials are materials or chemical substances having a size between 0–100 nm that display chain-breaking antioxidant activities [57–58]. The incorporation of nanomaterials and natural chain-breaking antioxidants is one of the most efficient strategies to combine the advantages of nanomaterials and natural antioxidants. For example, the natural phenolic compound gallic acid was covalently grafted on the surface of SiO_2_ nanoparticles. SiO_2_ nanoparticles provided thermal stability and chemical inertness while gallic acid provided chain-breaking antioxidant properties. By grafting antioxidant compounds on SiO_2_ nanoparticles, the deterioration can be decreased [59]. Similarly, gallic acid was covalently grafted on magnetite with an average size of 5 and 8 nm. The functionalization of ultrasmall magnetite with gallic acid increased free radical scavenging two- to fourfold compared to free magnetite [60]. Centurion et al. investigated the assembly of natural polyphenolic compounds triggered by liquid metals, leading to the formation of phenolic nanocoatings on substrates. In detail, nanocoatings of the phenolic compound pyrogallol deposited on polystyrene, glass beads, and paper yielded free radical scavenging activities of ca. 67%, ca. 84%, and ca. 92%, respectively [61].

**Superoxide dismutase (SOD) mimics:** SOD is a natural enzyme produced by organisms to protect themselves from oxidative stress and maintain normal physiological activity by neutralizing superoxide radicals. SOD degrades superoxide radicals into less harmful elements [62–64]. SOD is classified as a chain-breaking antioxidant because of its ability to directly react with ROS. However, SOD can also be considered as a preventive antioxidant because superoxide radicals can act as precursors to initiate peroxidation in some cases [65]. The main biochemical reactions of SOD relating to the decomposition of superoxide radicals into H_2_O_2_ and O_2_ occur at the active site. SOD is a promising antioxidant when it is used together with CAT and GPx for the decomposition of H_2_O_2_ into O_2_ and H_2_O.

SOD-mimicking nanomaterials have been synthesized to scavenge superoxide radicals. Encouraged by the discovery of the radical reaction of C_60_ fullerene in 1991 [66], C_60_ derivates were investigated for scavenging various free radicals including alkoxyl radicals, alkylperoxyl radicals, hydroxyl radicals, benzyl radicals, and superoxide radicals [67–69]. Since then, a large number of SOD-mimicking nanomaterials have been reported. Most of them employ transition metals such as iron [70], copper [71], cobalt [72], gold [73], manganese [74], platinum [75], or cerium [76] as main elements. Most transition metals have various oxidation states allowing them to generate cycles of redox reactions that are involved in superoxide radical scavenging. For example, cerium(IV) reacts with a superoxide radical to generate cerium(III) and oxygen. Then, cerium(III) reacts with the second superoxide radical to cerium(IV). The degradation of superoxide radicals by cerium nanomaterials can be described as follows:


[3]

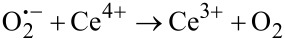




[4]

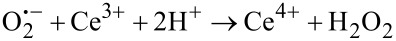



Cerium nanomaterials with higher Ce^4+^ content are known to have higher CAT activity, while cerium nanomaterials with greater Ce^3+^ content enhance SOD activity. It was reported that Ce^3+^ has more oxygen vacancies providing more active sites for the binding of superoxide radicals on the surface [77–79]. By controlling the Ce^3+^/Ce^4+^ ratio, the antioxidant activities of cerium nanomaterials can be flexibly designed to serve specific purposes.

Table 1 provides an overview of metal-based nanoantioxidants and their features.

**Table 1:** Summarization of metal-based nanoantioxidants and their remarkable features.

Nanomaterials (components)	Targets	Outcomes

PDA-Zn-BAI NPs (polydopamine, zinc, baicalein) [80]	H_2_O_2_, ABTS^•^, O_2_^•–^, HO^•^	* sustained release under acidic conditions (60.32% ± 3.19% over 5 days) and under normal conditions (36.67% ± 6.67% over 5 days)

CMNPs (ceria-coated melanin-PEG nanoparticles) [81]	H_2_O_2_, HO^•^, ONOO^–^, NO^•^	* achieved autoregenerative properties and outperformed aflibercept

AlOOH NPs (sol–gel-derived AlOOH NPs modified with natural phenolic acids) [82]	DPPH^•^	* exhibited good colloidal stability at wide pH range

MWCNTs (multi-walled carbon nanotubes, CNTs-COOH, CNTs@Fe_3_O_4_, CNTs@ZnFe_2_O_4_, CNTs@SiO_2_, CNTs@SiC) [83]	ABTS^•^, DPPH^•^, O_2_^•–^, HO^•^	* CNTs@SiO_2_ and CNTs@SiC inhibited the formation of peroxyl radicals

PEG-MeNPs (polyethylene glycol melanine nanoparticles) [84]	O_2_^•–^, H_2_O_2_, HO^•^, NO^•^, ONOO^–^	* significantly reduced the infarct area of the ischemic brain in a rat model of ischemic stroke

π-HBP (olefinic aliphatic AB_2_ monomer conjugating hyperbranched polymers) [85]	H_2_O_2_, HO^•^, O_2_^•–^	* self-assembly of π-HBP in aqueous solution formed 9.1 nm particles

FeO NPs (Iron oxide nanoparticles) [86]	DPPH^•^	* synthesized FeO NPs using *Amaranthus spinosus* leaf aqueous extracts

YGQDs (yellow luminescent graphene quantum dots) [87]	O_2_^•–^	* economic and scalable fabrication of YGQDs by using coal waste

CeO_2_ NPs modification with ligands, i.e. citrate ions, polysaccharides, phospholipid, protein [88]	O_2_^•–^, ROO^•^	* modification with ligand increased biocompatibility, SOD activity

PRA NPs (self-assembled poly(rosmarinic acid) nanoantioxidant) [89]	H_2_O_2_	* rapid liver-targeting capability

PluS-NO (Pluronic-silica nanoparticles) modification with nitroxide moieties [90]	ROO^•^	* each PluS-NO bore an average of 30 nitroxide units

EeNA (disulfide cross-linked albumin nanoparticle containing encapsulated edaravone) [91]	ROS	* largely accumulated in the liver and subsequently entered Kupffer cells within 60 min

### Application of metal-based nanoantioxidants in medicine and healthcare

#### Inflammatory diseases

Inflammation is a complex biological process occurring in response to harmful stimuli causing injury to healthy tissue. Oxidative stress resulting from a high level of ROS is one of the key contributors to inflammation [92–93]. Therefore, balancing the ROS level via antioxidant supplementation is a promising strategy for treating inflammatory diseases. Recently, Kim et al. introduced ultrasmall antioxidant cerium oxide nanoparticles (CeONPs) with strong SOD and CAT activities, which were used to decrease ROS levels and suppress the production of inflammatory cytokines (TNFα and IL-1β) in macrophages. CeONPs also decrease monocyte recruitment at inflammatory sites by balancing ROS levels [94]. Another modern strategy for developing anti-inflammatory nanodrugs is coupling transition metals with natural antioxidants. For example, Yuan et al. synthesized Fe-curcumin nanoparticles (Fe-Cur NPs) possessing intracellular ROS scavenging and anti-inflammatory capability for curing acute lung injury [95]. Fe-Cur NPs acted as an anti-inflammatory agent by downregulating the level of proinflammatory cytokines (TNF-α, IL-1β, and IL-6), suppressing NF-κB signaling pathways, inhibiting NLRP3 inflammasomes, and reducing intracellular Ca^2+^ in cells and tissues. One of the most important criteria of anti-inflammatory drugs is the direct delivery to the inflamed tissue [96–98].

To increase the targeting ability, anti-inflammatory agents can be wrapped with a cell membrane camouflage technique [99–101]. For example, Ma et al. constructed carbon dot-SOD nanozymes and CD98 CRISPR/Cas9 plasmids encapsulated in a metal-organic framework (MOF), and then camouflaged it with macrophage membrane [102]. In this system, the macrophage membrane guided the encapsulated nanozymes and CD98 CRISPR/Cas9 plasmids to accumulate at the inflammatory sites. The acidic environment at the inflammatory sites induced MOF disaggregation, resulting in the release of carbon dot-SOD nanozymes and CD98 CRISPR/Cas9 plasmids. The released nanozymes and plasmids contributed to ROS scavenging and reduced the CD98 gene expression, respectively. Another study employed CD44–hyaluronic acid interaction to endow a diselenide-bridged hyaluronic acid nanogel (SeNG) with the ability to specifically accumulate at CD44-overexpressed inflammatory cells [103]. Both in vitro and in vivo experiments demonstrated that the designed SeNG could not only eliminate ROS, but also up-regulated the Nrf2/HO-1 pathway to maintain redox homeostasis. Depending on the location of the accumulation, nanoantioxidants need to employ different encapsulating or coating strategies. Several studies used neutrophil membranes to increase the specificity of antioxidant delivery to ischemia-reperfusion (I/R) injury kidneys [104–105]. Neutrophils play a vital role in resolving inflammation, tissue repair, and initiating I/R-induced acute kidney injury progression. Moreover, I/R injury is characterized by a massive influx of neutrophils, which highly contribute to the pathophysiology of post-ischemic renal failure. Therefore, by coating with a neutrophil membrane, nanoantioxidants can be specifically delivered to kidneys with I/R injury.

A majority of reported anti-inflammatory nanomaterials mainly focus on the degradation of ROS. However, nanomaterials possessing the capability to degrade reactive nitrogen species (RNS) are rare. In response, Miao et al. synthesized polyethylene glycol-coated ultrasmall rhodium nanodots (Rh-PEG NDs) for scavenging both ROS and RNS in order to decrease inflammation [106]. Rh-PEG NDs exhibited O_2_^•–^, HO^•^, NO^•^, ONOO^–^, and H_2_O_2_ scavenging capability and were used to decrease the level of proinflammatory cytokines, including TNF-α and IL-6, at the inflammatory sites. As another example, an alendronate-coated nanoceria (CeAL) nanozyme was reported by Zhou et al. for both ROS and RNS scavenging [107]. Other RNS scavenging nanomaterials such as Co-doped Fe_3_O_4_ nanozymes [108], WS_2_, MoSe_2_, WSe_2_ nanosheets [109], carbogenic nanozymes [110], and Pt/CeO_2_ nanozymes [111–112] also showed excellent RNS scavenging capability.

Table 2 gives an overview of experiments using antioxidant nanomaterials for the treatment of oxidative stress-related diseases.

**Table 2:** In vitro and in vivo experiments using antioxidant nanomaterials for the treatment of oxidative stress-related diseases.

Nanomaterial	Coating strategy	Location of accumulation	Evaluation model	Function/Effect

N–Cu_5.4_O@DFO NPs [104]	neutrophil membrane	ischemia/reperfusion injury kidney	- in vitro in HEK293 cells - in vivo in female BALB/c mice	- high biocompatibility, stability, and antioxidant activity - reduced oxidative stress - reduced inflammatory response - achieved synergistic therapy against renal ischemia/reperfusion injury
poly(rosmarinic acid) nanoantioxidant (PRA NP) [89]	none	liver	- in vivo in male BALB/c mice and nude mice	- glycerol monooleate induced self-assembly poly(rosmarinic) into nanomedicines - PRA NPs had high therapeutic effects in the in vivo treatment of acute liver injury through increasing antioxidant activity
two-dimensional ceria nanoplates [32]	none	ischemia/reperfusion injury brain tissue	- in vitro in SH-SY5Y cells - in vivo in male Sprague-Dawley rats and C57 mice	- 1.2 nm ultrathin nanoplates - ca. 2.6-fold higher SOD activity than narutal SOD - high efficiency in the treatment of reperfusion-induced injury in ischemic stroke - low toxicity
Au@Cu_2_O [48]	none	A549 cells and zebrafish	- in vitro in A549 cells and zebrafish	- achieved complete antioxidant system through scavenging H_2_O_2_, HO, O_2_^•–^ - mimicked CAT, SOD, GPx, and POD activities
diselenide-bridged hyaluronic acid nanogel (SeNG) [103]	hyaluronic acid (HA)	colitis tissues	- in vitro in RAW 264.7 and HT29 cells - in vivo in male BALB/c mice	- SeNG accumulated in colitis tissues through CD44–HA interaction - SeNG effectively eliminated ROS - SeNG up-regulated Nrf2/HO-1 pathway to maintain redox homeostasis - high anti-inflammatory effect in an acute colitis mouse
neutrophil-like cell-membrane-coated mesoporous Prussian blue nanozyme (MPBzyme@NCM) [113]	neutrophil membrane	Ipsilateral brain	- in vitro in Human HL-60 cells - in vivo in male C57/BL6 mice	- noninvasive active-targeting therapy - long-term in vivo therapeutic efficacy for ischemic stroke
mesoporous silica nanoparticles coated with caffeic acid (MSN-CAF) or rutin (MSN-RUT) [114]	caffeic acid, rutin	Caco-2 and the HaCaT cell lines	- in vitro in intestinal Caco-2 and the epidermal HaCaT cell lines	- reduced ROS level after 24 h - activated Nrf2 antioxidant pathway - high cellular viability

#### Neurological diseases

**Figure 3:**
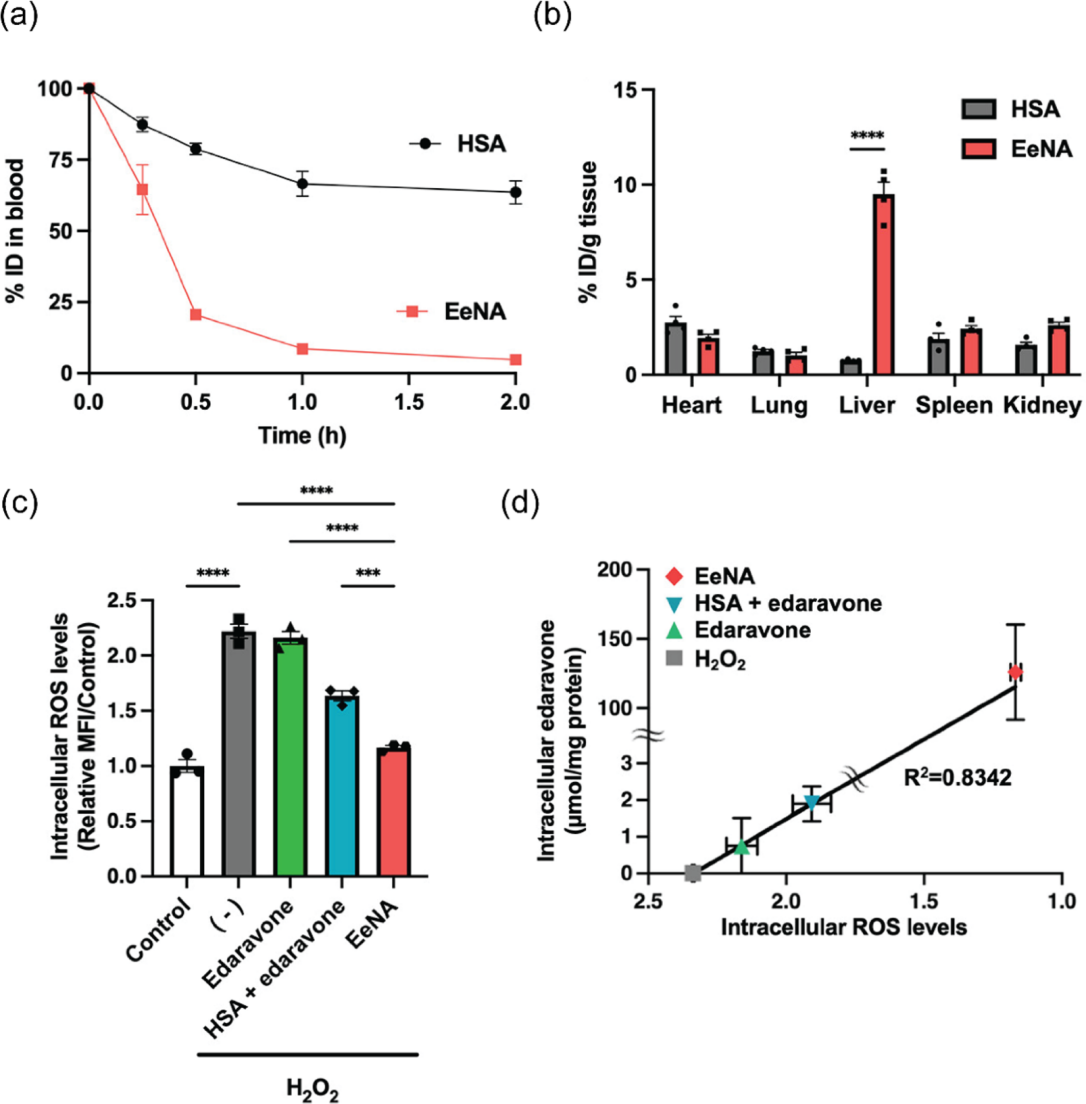
(a) Percent injected dose (%ID) in blood of EaNA and Cy5-labeled human serum albumin (HSA). (b) Organ distribution of EeNA and HSA. Data are represented as the mean ± s.e.m. (*n* = 4). ****p < 0.0001 vs HSA. (c) Comparison of ROS scavenging activity between EeNA, HSA + edaravone, and edaravone. Data are represented as the mean ± s.e.m. (*n* = 3). ***p < 0.001, ****p < 0.0001. (d) Correlation between intracellular ROS levels and intracellular edaravone. Figure 3 was adapted with permission from [91]. Copyright 2023 American Chemical Society. This content is not subject to CC BY 4.0.

Overproduction of ROS and RNS in the brain is involved in neurological diseases such as Parkinson’s disease, Alzheimer’s disease, traumatic brain injury, and stroke. Neurological diseases are recognized as a major threat to human health and are considered incurable diseases because most drugs cannot cross the blood–brain barrier (BBB) [115–116]. Besides, the accumulation of drugs at damaged areas of the BBB can lead to an unprotected, disrupted BBB and to disturbances of the brain microenvironment. In contrast, the integrity of the BBB can decrease the accumulation of drugs in brain lesions. This dilemma is a big challenge in the development of an effective treatment for neurological diseases. Antioxidant nanomaterials with excellent ROS and RNS scavenging are expected to become a promising solution for curing neurological diseases because their surface can be modified, which endows them with the ability to cross BBB. Cerium oxide nanoparticles with high antioxidant activity make them a possible medication for alleviating neurological diseases. Along with significant advances in nanotechnology, Fu et al. synthesized cerium oxide with an ultrasmall size of 2 nm possessing outstanding ROS scavenging and BBB crossing ability almost without negative effects in vitro and in vivo [117]. Nanoceria loaded with edaravone and coated with angiopep-2 and poly(ethylene glycol) on their surface have been used for stroke treatment [118]. This nanocomposite system consisted of three key components: (1) edaravone and nanoceria were responsible for ROS scavenging; (2) poly(ethylene glycol) increased biocompatibility, monodispersity, and extended the half-life in the bloodstream; and (3) angiopep-2 served as targeting ligand, which specifically binds to the low-density lipoprotein receptor-related protein overexpressed on cells that comprise the BBB. Figure 3 illustrates the biodistribution and ROS scavenging activity of edaravone-encapsulated nanospherical albumin (EeNA) [91].

Alzheimer’s disease is a neurological disease that slowly destroys thinking skills and memory [119]. The main pathogenic mechanisms of Alzheimer’s disease involve not only ROS overproduction but also amyloid beta (Aβ) fibril accumulation. Liu et al. reported a nanosystem employing polydopamine and ruthenium (PDA-Ru) as key elements for ROS scavenging and decomposition of mature Aβ fibrils [120]. PDA-Ru nanoparticles could degrade Aβ fibrils under low-power laser irradiation because of their great photothermal effect. Moreover, PDA-Ru nanoparticles could decompose H_2_O_2_ owing to their strong CAT activity. PDA-Ru nanoparticles effectively improved memory capacity and decreased neuroinflammation in APP/PS1 mice. Another research used Cu*_x_*O@EM-K as a stabilizer for therapeutic agents for peripheral Aβ clearance [121]. The Cu*_x_*O@EM-K consisted of a Cu*_x_*O core wrapped by a modified erythrocyte membrane with Aβ-specific ligand KLVFF. KLVFF worked together with the erythrocyte membrane to selectively capture Aβ in the blood. At the same time, the Cu*_x_*O core exhibited multiple antioxidant activities mitigating Aβ-induced membrane oxidative damage and stabilizing the outer erythrocyte membrane.

#### Anti-aging

Aging is a natural process defined as a time-related deterioration of the physiological functionalities that are necessary for fertility and survival [122]. The free radical theory of aging proposes that aging is caused by the accumulation of ROS during the metabolism. Although the natural antioxidant defense systems in the human body including CAT, SOD, and GPx can neutralize ROS, the system cannot completely stop the aging process [7,123]. In this context, antioxidant supplementation is regarded as an efficient strategy to defend humans against aging. Natural compounds from plant-derived extracts such as polyphenols, tocopherols, carotenoids, and ascorbic acid are the most important and common ingredients of antioxidant supplements. However, these compounds have low bioavailability related to low stability, solubility, and absorbance in the gastrointestinal tract [124–127]. Recently, with extraordinary progress in nanotechnology development, nanodelivery systems have been considered as an effective solution because of their ability to overcome the physical and chemical barriers in the gastrointestinal tract. Such barriers are the intestinal mucosal barrier, acidic conditions in the stomach, and selectively permeable membranes of enterocytes. Moreover, natural compounds delivered by nanocarriers have various therapeutic advantages such as no or minimized side effects, long storage life, enhanced residence time, extended circulation time, increased half-life, and decreased dose [128–131].

Nanodelivery systems for natural antioxidants can be divided into two main classes, namely natural and synthetic nanocarriers. Because of the advantages of synthetic nanocarriers regarding easier customization of size, surface properties, charge, and morphology, we will focus on synthetic nanocarriers. Various types of synthetic nanocarriers have been developed to deliver natural antioxidants: (1) nanoliposomes, which are a nanoscale bilayer lipid vesicle [132]; (2) nanocapsules, which consist of an inner aqueous core surrounded by a nontoxic polymeric membrane [133]; (3) solid lipid nanoparticles, which consist of a solid lipid core stabilized by a surfactant [134]; and (4) nanocrystals, which are a cluster of hundreds to thousands atoms aggregated together [135]. The potential of natural antioxidant nanodelivery systems for treating age-related metabolic disorders has been proved in both in vivo and in vitro studies. For example, a nanoparticle-based formulation of curcumin exhibited better anti-aging properties than natural curcumin. By encapsulating curcumin in nanocarriers or by conjugating it to metal oxide nanoparticles, the solubility and bioavailability of curcumin have been substantially improved, leading to a rise in its pharmacological efficiency [136–139]. Pharmacokinetic analysis of curcumin-loaded polymeric nanoparticles after oral delivery in mice demonstrated a 20-fold decrease in dose requirement compared to natural curcumin [140]. Both experimental and molecular dynamics simulation studies suggested an optimal ferulic acid (an antioxidant in plants) concentration of 0.5 wt % for the successful formation of ferulic acid-loaded lipid-based nanoparticles [141]. The ferulic acid-loaded nanoparticles with improved bioavailability can be useful for skin care products and human skin cancer treatment [142–144].

#### Wound repair

The skin is the outermost layer, the largest organ, and the first barrier protecting our body against toxic elements, infections, and dehydration, which makes it vulnerable. A major skin injury can cause severe problems to human health such as increased risk of infections, dehydration, and immune system disorders [145–146]. Wound repair is a crucial process for the recovery of injured skin by which the integrity of the wounded area is restored and regenerated. In wound repair, ROS acts as a double-edged sword because ROS have both positive and negative effects on wound repair. At a low ROS level, the wound repair process benefits from ROS because ROS can prevent microbial infections. However, a high level of ROS increases the deleterious effects on wound repair by increasing the inflammation [147–148]. Therefore, nanomaterials with antioxidant effects are proposed to balance the ROS level in the wound repair process. As a typical example, nanofibers composed of polygalacturonic acid, hyaluronic acid, and embedded silver nanoparticles were applied to recover wounded areas of albino rats in vivo [149]. In this nanocomposite, silver nanoparticles acted as an antioxidant, polygalacturonic acid acted as a reducing agent for generating silver nanoparticles from silver ions, whereas hyaluronic acid enhanced hydrophilicity and strain activities.

**Figure 4:**
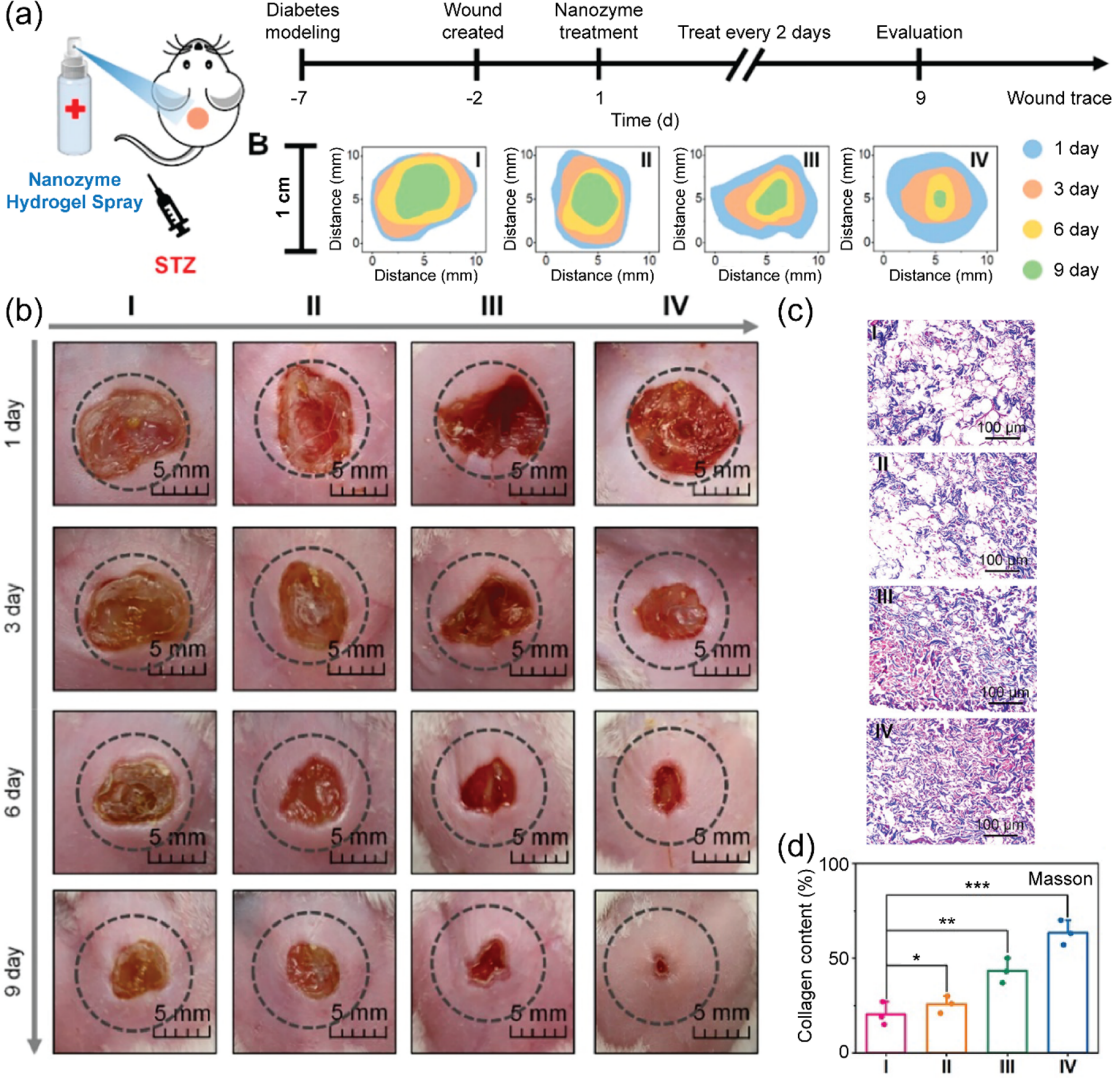
In vivo wound healing of Au/Cu_1.6_O/P–C_3_N_5_/Arg@HA. (a) Schematic illustration of using Au/Cu_1.6_O/P–C_3_N_5_/Arg@HA for treating a diabetic mouse wound model infected with Streptozotocin (STZ). (b) Traces of wound closure within nine days after four different treatments (I: control, II: ultrasound, III: Au/Cu_1.6_O/P–C_3_N_5_/Arg@HA, and IV: Au/Cu_1.6_O/P–C_3_N_5_/Arg@HA + ultrasound). (c, d) Masson staining of wounded tissues after four different treatments (I, II, III, and IV). Figure 4 was adapted with permission from [162]. Copyright 2023 American Chemical Society. This content is not subject to CC BY 4.0.

To enhance wound repair, metal or metal oxide-based nanoantioxidants can be conjugated with miRNA146a. Because of the capability of miRNA146a to downregulate IL-6 and IL-8 expression, this combinational approach decreases both ROS levels and inflammation [150–151]. Moreover, though excellent ROS scavenging, metal and metal oxide effectively protect miRNA146a from oxidative damage [152–153]. However, intradermal injection is required to deliver the antioxidant nanocomposites into the body, which is not an ideal method because it can be painful. In response, antioxidant nanomaterials coupled with hydrogels provide a promising method to topically deliver antioxidant nanomaterials into the body [154–155]. Nanoantioxidants (such as fullerene, cerium, gold, silver, and iron nanoparticles) and hydrogels (such as gelatin methacryloyl, chitosan/polycaprolactone, polyvinyl alcohol/chitosan, and polypyrrole-grafted gelatin) were combined to generate diverse combinations of nanocomposites for wound repair [156–161]. This strategy not only endows nanoantioxidants with topical delivery but also enhances stability and biocompatibility. Recently, Shang et al. used a Au/Cu_1.6_O/P–C_3_N_5_/Arg@HA nanocomposite hydrogel spray coupled with ultrasound for diabetic wound healing (Figure 4) [162]. This nanocomposite spray exhibited five types of enzyme-like activities, that is, CAT-, SOD-, peroxidase (POD)-, glucose oxidase (GOx)-, and nitric oxide synthase (NOS)-like activities. More interestingly, both in vitro and in vivo experiments demonstrated that the nanocomposite spray can be activated by the diabetic foot ulcer microenvironment to accelerate diabetic wound healing through decreasing inflammation, lowering blood glucose, relieving hypoxia, promoting angiogenesis, and eliminating pathogenic bacteria. Other in vivo experiments demonstrated that mice treated with silver-based nanocomposites showed significantly higher (*p* < 0.001) wound contraction compared to saline solution (negative control), and the wound was healed after 11 days [163].

#### Cancers

**Figure 5:**
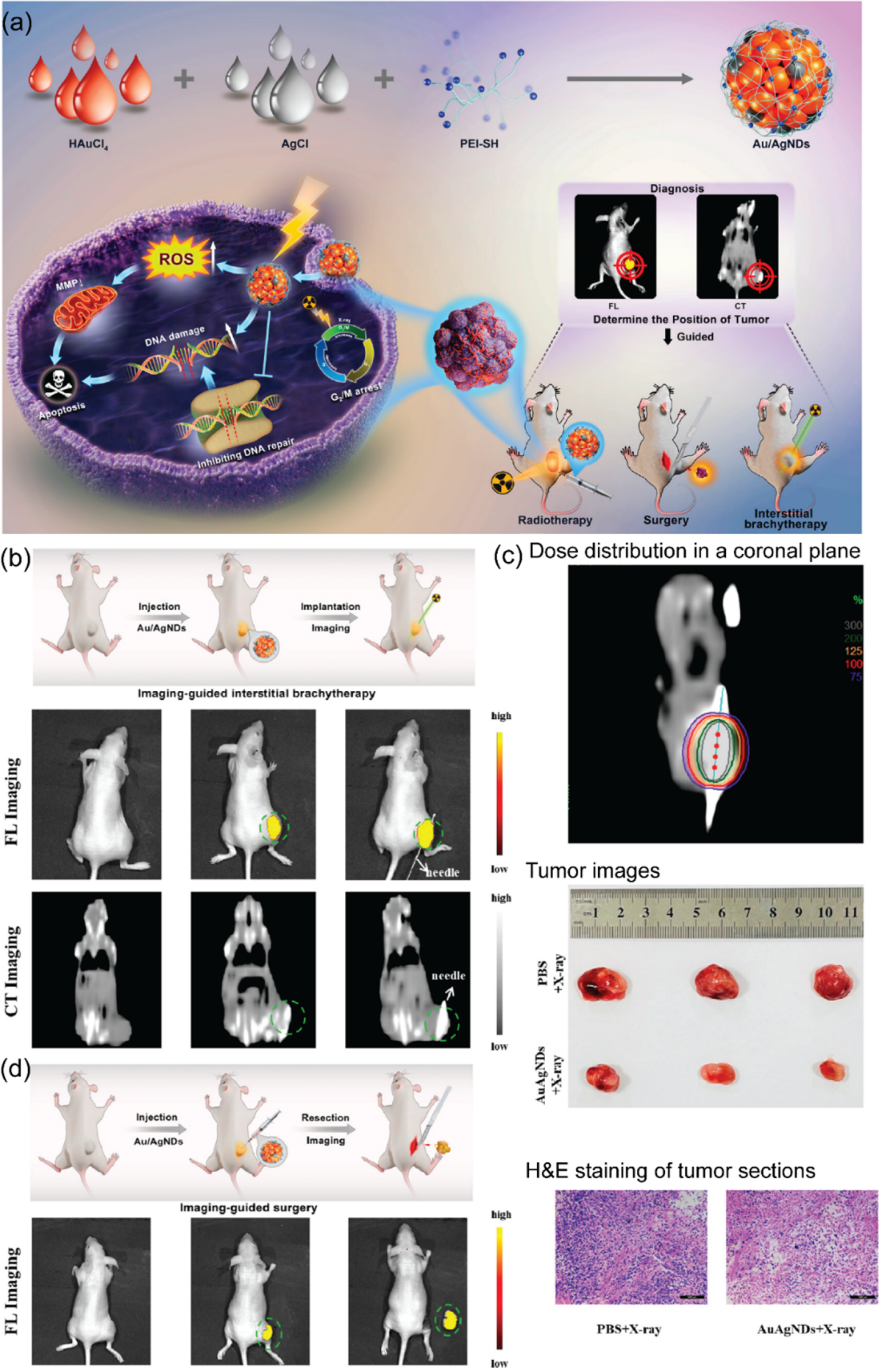
Multifunctional theranostic platform using Au/Ag nanodots for the clinical diagnosis and treatment of cancer. (a) Schematic illustration of synthesis of Au/AgNDs used for dual-mode imaging-guided surgery, enhanced radiotherapy, and brachytherapy of tumors. (b) Fluorescence/computed tomography (FL/CT) dual-mode imaging guided interstitial brachytherapy and FL imaging-guided tumor resection. (c) Dose distribution in a coronal plane for an interstitial brachytherapy plane. Tumor images collected from mice with different treatment groups. H&E staining of primary tumor sections after different treatments (scale bar: 100 μm). (d) Schematic illustration of intratumoral injection of Au/AgNDs and fluorescence-mediated tumor resection. Fluorescence image (control group), FL image of the subcutaneous HeLa tumor-bearing nude mice model post injection of Au/AgNDs, and FL imaging-guided surgical removal of tumor. Figure 5 was adapted from [170] (© 2023 Z. Wang et al., published by American Chemical Society, distributed under the terms of the Creative Commons Attribution 4.0 International License, https://creativecommons.org/licenses/by/4.0).

Cancer is considered one of the most common causes of death worldwide and has become a major health concern. Cancer involves abnormal cells that grow uncontrollably and can spread to other parts of our body. The initiation of cancer relates strongly to high levels of ROS that can cause damage to DNA molecules resulting in abnormal cell proliferation. Various studies reported that oxidative damage can have a deleterious effect on cancer development through raising genetic mutations, abnormal protein functions, and tumor growth [164–165]. Antioxidants contribute to cancer inhibition and cancer treatment by several mechanisms. First, nanoantioxidants reduce cancer initiation by protecting DNA molecules from oxidative stress and stimulating DNA repair. For example, platinum nanoparticles inhibited the growth of epithelial lung cancer cells by enhancing SOD, GPx, and CAT activities [166]. Second, nanoantioxidants can be used to support cancer therapies such as photodynamic therapy (PDT) and photothermal therapy (PTT) [167]. In this strategy, nanomaterials with antioxidant activities enhance PDT and PTT efficacy by reducing hypoxia in the tumor sites. In this regard, nanomaterials exhibit CAT-like activity with the ability to generate O_2_ from H_2_O_2_ to increase the performance of PDT and PTT in tumors. For example, nano-MnO_2_ with acid-/redox-responsive properties could decompose acidic H_2_O_2_ at the tumor sites by exhibiting CAT activity [168]. Similarly, Zeng et al. employed this strategy to fabricate a CeO_2_ nanozyme-loaded nanovesicle to address hypoxia at the tumor sites [169]. In this system, the CeO_2_ nanozyme with the ability to generate the cycle of redox reactions continuously provides a significant amount of O_2_ for assisting cancer treatments. Recently, Wang et al. developed multifunctional Au/Ag nanodots (Au/AgNDs) as “pilot light” for real-time guided surgery (Figure 5) [170]. The authors proved that Au/AgNDs enhanced the deposition of ionizing radiation energy, increased intracellular ROS generation, and significantly improved radiotherapy efficacy. Also, using Au/AgNDs for fluorescence images could distinguish tumors from normal tissue, thus, assisting surgeons during tumor resection, which is a novel strategy and vision for the clinical diagnosis and treatment of cancer.

#### Cardiovascular diseases

Cardiovascular diseases (CVDs) account for many disorder conditions of blood vessels and heart. Atherosclerosis, a chronic inflammatory disease characterized by the buildup of plaque within arterial walls, is a major factor in strokes and myocardial infarctions and remains a substantial global health challenge. In recent years, researchers have explored innovative approaches for the treatment of atherosclerosis, with a particular focus on the use of metal-based nanomaterials. The advancement of nanomedicines in CVD treatments will gain from the comprehensive understanding of the rate and extent of nanoparticles (NPs) inside the atherosclerotic plaques [171].

One promising avenue involves the development of metal-based NPs for targeted drug delivery to atherosclerotic lesions. These NPs, often composed of biocompatible metals such as gold, silver, or iron, offer unique properties that enable precise drug delivery to affected areas while minimizing systemic side effects. For instance, studies by Cheng et al. revealed the successful application of gold NPs for delivering anti-inflammatory agents to atherosclerotic plaques and tomography imaging of atherosclerosis [172]. Recent research by Mao et al. and Wang et al. highlighted the efficacy of a bioresorbable magnesium alloy stent coated with an anti-proliferative drug, offering a dual benefit of mechanical support and localized drug release, leading to improved outcomes in atherosclerosis treatment [173–174]. Besides, since zinc has emerged as a promising candidate because of its anti-inflammatory and antioxidant effects, a study by Chen et al. explored the use of Zn NPs to modulate macrophage polarization within atherosclerotic plaques, proving the potential for metal-based materials to actively influence the underlying inflammatory processes [175]. Clinical applications of metal-based nanomedicines, particularly AuNPs, have been implemented in various settings, with twelve clinical trials already completed or in progress. Notably, one of these trials, NCT01436123, focuses on utilizing heat for the photothermal treatment of atherosclerosis [176]. In an observational study with three arms, Kharlamov et al. investigated 180 patients diagnosed with coronary artery disease (CAD). Their findings unveiled a notable regression of coronary atherosclerosis associated with plasmonic photothermal therapy using silica–gold NPs (SiO_2_-AuNPs) [177].

Metal-based NPs exhibit the ability to scavenge free radicals, preventing oxidative stress and cellular damage thanks to their diverse size and surface properties. Administering platinum NPs to transient middle cerebral artery occlusion (tMCAO) mice significantly decreased the infarct volume, matrix metalloproteinase-9 (MMP-9) activation, and the generation of superoxide anions (•O^2−^) [178]. Titanium dioxide NPs have also gained attention for their ability to reduce oxidative stress and inflammation in atherosclerosis [179–180]. The incorporation of these metal-based nanomaterials into therapeutic strategies signifies a paradigm shift towards personalized and targeted treatments for sclerosis. Recently developed Fe_3_O_4_–CeO_2_ core–shell NPs have shown great potential as platforms for both the diagnosis and treatment of vascular disorders associated with ROS. This is attributed to their impressive magnetic resonance imaging (MRI) capabilities and effective scavenging ability against ROS [181]. Additionally, it has been reported that biocompatible covalently doped carbon dots (CuZn-CDs) with both CAT and SOD functionalities demonstrate significant in vitro capability to scavenge ROS. These carbon dots exhibit a protective effect on cardiomyocytes, guarding against damage induced by ROS and providing cardio protection during ischemia-reperfusion injury [182].

Metal-based NPs have also shown promising potential in the treatment of myocardial ischemia and injury, offering innovative approaches to address cardiovascular challenges. In 2019, Zhang and collaborators synthesized mesoporous carbon nanospheres (PMCSs) derived from a MOF precursor, exhibiting dual photodynamic and photothermal characteristics. Utilizing this framework, concentration and temperature at thrombotic sites were elevated significantly upon local irradiation (808 nm laser), resulting in the disruption of blood clots [183]. Currently, therapies for myocardial ischemia-reperfusion injury involve the utilization of Fe_3_O_4_, CeO_2_, Au, and Cu as highlighted in the study by Baldim and co-workers [184]. Notably, CeO_2_ exhibits extensive antioxidant activities attributed to the redox cycling between cerium(IV) and cerium(III). SOD activity immobilized through cross-linking on a Zr framework (SOD-ZrMOF) demonstrates effective scavenging of ROS and inhibition of oxidative stress. A prolonged investigation using an animal model of acute myocardial infarction revealed that SOD-ZrMOF has the capacity to diminish the infarct area and preserve cardiac function [185].

NP scaffolds, capable of controlled degradation over time, provide temporary mechanical support while facilitating tissue healing and implantation. The presence of AuNPs in infarcted heart tissues has been associated with a reduction in infarction size, TNF-α levels, cardiac fibrosis, and improvement in cardiac systolic function. Gold nanorods serving as surface-enhanced Raman scattering probes have demonstrated sensitivity for the early detection of ICAM-1, a significant signal for screening atherosclerosis, particularly in macrophages. Moreover, AuNPs with different sizes and shapes exhibit the capability to inhibit angiogenesis, especially at 20 nm size [178,186]. In 2022, García-Rubio and colleagues introduced a novel diagnostic approach to differentiate between normal blood pressure and hypertension. This method involves the conjugation of gold nanoparticles (AuNPs) with an anti-epithelial sodium channel (ENaC), known as a marker for arterial hypertension found in membrane platelets [186]. Beyond nanotechnology, the field has witnessed developments in metallic biomaterials for vascular tissue engineering. Titanium alloys, owing to their favorable mechanical properties and biocompatibility, have been explored for the fabrication of vascular grafts highlighting their potential as a durable and biocompatible alternative in atherosclerosis-related revascularization procedures [187]. Similarly, studies conducted by Li et al. demonstrated that biodegradable Mg scaffolds have shown promise in promoting vascular regeneration [188].

In brief, the use of metal-based nanomaterials in CVD treatment encompasses a range of innovative approaches from targeted drug delivery using NPs to the development of advanced metallic biomaterials for vascular interventions and cardiovascular therapeutics. As researchers continue to discover the intricacies of CVDs, the integration of metal-based materials holds promise for more effective clinical strategies.

### Challenges of metal-based nanoantioxidants in medicine and healthcare

Metal-based nanoantioxidants, inheriting excellent properties of nanomaterials such as large specific surface area, high catalytic activity, and tunable structure and composition, have great potential for providing effective and safe treatment for oxidative stress-related diseases. However, regarding the translation from clinical research to clinical practice, some limitations of metal-based nanoantioxidants need to be overcome. First, the nanoscale size of metal-based nanoantioxidants makes these materials distinct from bulk materials because of the quantum size effect. The uncommon nanoscale sizes, which are not familiar to biological systems can cause detrimental effects on human health. Sobolewski et al. confirmed the Fe_2_O_3_ nanoparticles with sizes between 11.2 and 13.6 nm can lead to oxidative damage and neurotoxicity in the mouse brain [189]. Meanwhile, the rapid clearance of SiNPs from blood depletes plasma apolipoprotein A-I and facilitates atherosclerosis [190]. Si nanoparticles were reported for their ability to invade the cytoplasm, modify the intracellular microstructure, and promote inflammatory reactions through activating NLRP3 inflammasomes [191]. Also, in an animal experiment, SiO_2_ nanoparticles induce thyroid hormone disruption in zebrafish larvae co-exposed to tetrabromobisphenol A by promoting its bioaccumulation and bioavailability [192]. Xu et al. demonstrated that TiO_2_ nanoparticles can promote damage to adipose derived stromal cells at low concentrations [193]. Accordingly, the surface chemistry of nanosized metals plays a major role in their toxicity. The toxicity of nanosized metal-based antioxidants can be divided into chronic toxicity and acute toxicity. Chronic toxicity is caused by long-term exposure to nanosized metal-based antioxidants, facilitating organ damage and cancers, while acute toxicity causes tissue damage immediately after exposure to certain nanosized metals.

Besides, the large specific surface area of nanomaterials has some benefits regarding excellent ROS scavenging; yet, a large specific surface area can also be a double-sided sword because it increases nano–bio interactions leading to toxic effects. Therefore, health and safety profiles of metal-based nanoantioxidants need to be provided. Second, metal-based nanoantioxidants have very diverse shapes, sizes, and surface functional groups. However, modifying these factors significantly changes the way metal-based nanoantioxidants affect to immune system. It was reported that nanomaterials can either enhance or suppress the immune system [194]. Therefore, the effect of each factor on the immune system needs to be fully investigated. Third, metal-based nanoantioxidants face the same biological and physiological barriers as other medicines, which significantly limits their applications in clinical practice. Some common barriers include blood–brain barrier, gastrointestinal barrier, cellular membrane, and blood vessel fenestration [195]. Although scientists have made significant effort in solving this problem, only a few metal-based nanoantioxidants successfully cross these barriers.

## Conclusion and Future Perspectives

The consumption of large amounts of fat and a sedentary lifestyle have resulted in an excess of ROS production in the human body resulting in oxidative stress, which is subsequently responsible for harmful diseases such as cancers, Alzheimer’s disease, and Parkinson′s disease. Although natural antioxidants from plants and animals play an important role in overcoming oxidative stress, such antioxidants have several limitations such as low stability, difficult long-term storage, and high cost of large-scale production. Fortunately, with the progress in nanotechnology, we can design nanomaterials exhibiting antioxidant activities that overcome the limitations of natural antioxidants. The 21st century has witnessed significant development in metal-based nanoantioxidants with a large number of publications. Based on the mechanism of action, metal-based nanoantioxidants can be divided into two main types, namely (1) preventive metal-based nanoantioxidants and (2) chain-breaking metal-based nanoantioxidants. Preventive metal-based nanoantioxidants can interfere with the initiation of oxidative reactions, while chain-breaking metal-based nanoantioxidants stop the oxidative reactions after they begin. The strategy for the fabrication of antioxidant nanomaterials is to mimic natural antioxidant enzymes such as CAT, GPx, and SOD. With excellent properties such as high stability, bioavailability, and activity, metal-based nanoantioxidants are widely applied in medicine and healthcare. Metal-based nanoantioxidants have been repeatedly proven to improve oxidative stress-related diseases such as inflammatory diseases, neurological diseases, and cancers; also, they showed activity in wound repair and anti-aging treatments.

Although the past decade has witnessed extraordinary progress in developing nanoantioxidants, further research is still required to overcome the limitations of metal-based nanoantioxidants and to bring this technology closer to clinical practice. Future research needs to focus on the toxicology of metal-based nanoantioxidants and the reduction of detrimental effects of these nanomaterials on human health. Surface modification can become an effective method to decrease the toxicity of nanomaterials through coating with biocompatible polymers such as chitosan, polydopamine, and polyethylene glycol. From this perspective, bio-inspired nanoantioxidants could be a potential solution for safety issues. Furthermore, metal-based nanoantioxidants are still in the early stage of clinical practice and face the issue of less accurate accumulation at a specific site. In the next stage, a deep and wide collaboration between chemistry, biomedical fields, nanomaterials science, and physics is needed to accurately control the shape, size, and morphology of metal-based nanoantioxidants for more specific accumulation.

## Data Availability

All data that supports the findings of this study is available in the published article and/or the supporting information to this article.
